# Diet and Kidney Function: a Literature Review

**DOI:** 10.1007/s11906-020-1020-1

**Published:** 2020-02-03

**Authors:** A. C. van Westing, L. K. Küpers, J. M. Geleijnse

**Affiliations:** Division of Human Nutrition and Health, Wageningen University, Stippeneng 4, 6708 WE Wageningen, The Netherlands

**Keywords:** Dietary patterns, Foods, Beverages, Kidney function, Chronic kidney disease, Prospective cohort studies, Glomerular filtration rate

## Abstract

**Purpose of Review:**

The burden of chronic kidney disease (CKD) is increasing worldwide. For CKD prevention, it is important to gain insight in commonly consumed foods and beverages in relation to kidney function.

**Recent Findings:**

We included 21 papers of prospective cohort studies with 3–24 years of follow-up. We focused on meat, fish, dairy, vegetables, fruit, coffee, tea, soft drinks, and dietary patterns. There was convincing evidence that a healthy dietary pattern may lower CKD risk. Plant-based foods, coffee, and dairy may be beneficial. Unhealthy diets and their components, such as red (processed) meat and sugar-sweetened beverages, may promote kidney function loss. For other foods and beverages, associations with CKD were neutral and/or the number of studies was too limited to draw conclusions.

**Summary:**

Healthy dietary patterns are associated with a lower risk of CKD. More research is needed into the effects of specific food groups and beverages on kidney function.

**Electronic supplementary material:**

The online version of this article (10.1007/s11906-020-1020-1) contains supplementary material, which is available to authorized users.

## Introduction

Chronic kidney disease (CKD) is a major public health burden [1, 2], with a global prevalence of ~ 11% in the general adult population [1]. If left untreated, CKD slowly progresses to end-stage renal disease, which requires dialysis or kidney transplant [2, 3]. CKD is bidirectionally associated with cardiovascular diseases (CVD) [4, 5]. Hypertension [6] and type 2 diabetes mellitus (T2DM) [7, 8] are independent risk factors for CKD [6, 7], and their global prevalences are increasing [9, 10], which will likely impact CKD. Worldwide, a 31.7% increase of CKD mortality was observed over the last decade [11].

Lifestyle factors, including smoking [12], alcohol use [13], and physical inactivity [14], could promote CKD. Apart from that, there is increasing scientific interest in the potential role of diet [15, 16]. High salt intake is an established risk factor for kidney function decline [17, 18], mainly through its adverse effect on blood pressure and vascular health [19–21]. Less is known about other dietary factors. Therefore, we reviewed the current evidence on foods, beverages, and overall dietary quality in relation to the risk of incident CKD using data from prospective cohort studies.

## Methods

We performed a comprehensive search in PubMed of papers published until August 2019 describing prospective cohort studies, supplemented by manual searches of reference lists from appropriate studies. The review is based on prospective cohort studies with at least 3 years of follow-up that reported on the relation between food groups, beverages, and dietary patterns and kidney function in populations free from CKD (defined as mean estimated glomerular filtration rate (eGFR) > 60 ml/min/1.73 m^2^).

Foods of interest were red (processed) meat, poultry, fish, dairy, vegetables, legumes, nuts, and fruits. Beverages included coffee, tea, sugar-sweetened beverages (SSBs), and diet beverages. Dietary patterns included adherence to the Dietary Approach to Stop Hypertension (DASH) diet, Mediterranean diet, and other healthy dietary patterns. Unhealthy diets were high fat, high sugar diets, and diets with a high acid load.

Concerning kidney function, we selected studies with data on the eGFR, derived from the Chronic Kidney Disease Epidemiology Collaboration (CKD-EPI) equation [22, 23] and Modification of Diet in Renal Disease (MDRD) [24].

Reasons for exclusion of articles were studies with (1) follow-up less than 3 years, (2) study design other than prospective cohort study, (3) study population with T2DM and analgesic use, (4) no full-text available, and (5) focus on end-stage renal disease. The selection process is shown in eFig. 1.

From selected papers, we extracted data on population characteristics, study design, intakes of foods and/or beverages, kidney function outcomes, risk estimates for diet-kidney function associations, and potential confounders.

The primary outcome for this review was “incident CKD” based on eGFR cutoff criteria, described in eTable 1. Associations between foods, beverages, and incident CKD in different studies were expressed as odds ratios (OR), obtained from logistic regression analysis, or hazard ratios (HR), obtained from Cox proportional hazard analysis with corresponding 95% confidence intervals (CI). In this review, OR and HR are both denoted as relative risks (RRs). Continuous associations between food groups, beverages, and change in eGFR are expressed as beta regression coefficients, obtained from multivariable linear regression.

RRs and betas from fully adjusted models are reported in tables with potential confounders. When these models included possible intermediates (i.e., factors could play a role in the biological pathway), risk estimates from less adjusted models are given. Two-sided *P* values < 0.05 for risk estimates were considered statistically significant.

## Results

**Fig. 1:**
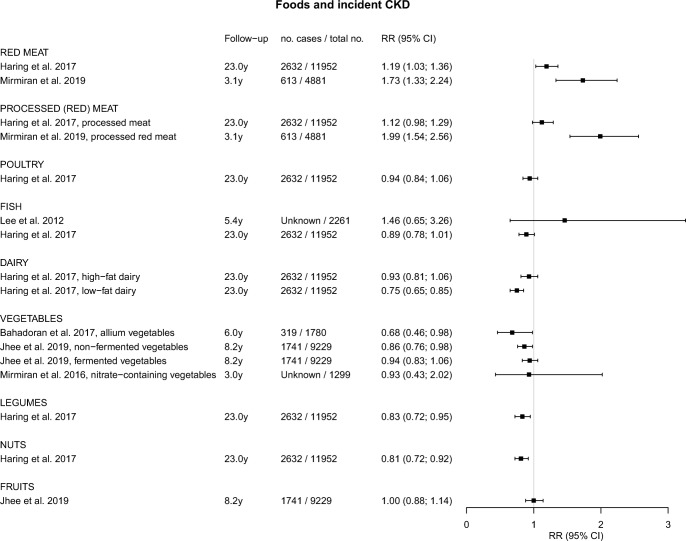
Forest plot for associations between commonly consumed foods and incident chronic kidney disease

**Fig. 2:**
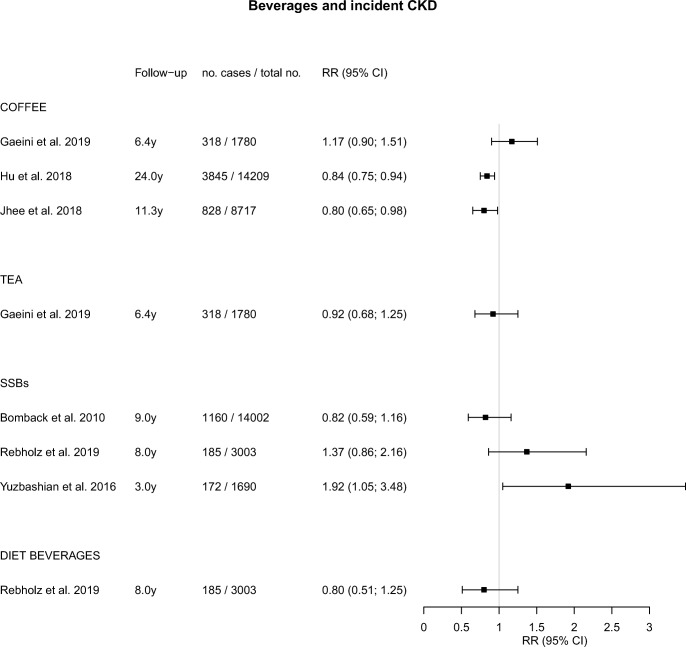
Forest plot for associations between commonly consumed beverages and incident chronic kidney disease. SSB, sugar-sweetened beverages

**Fig. 3:**
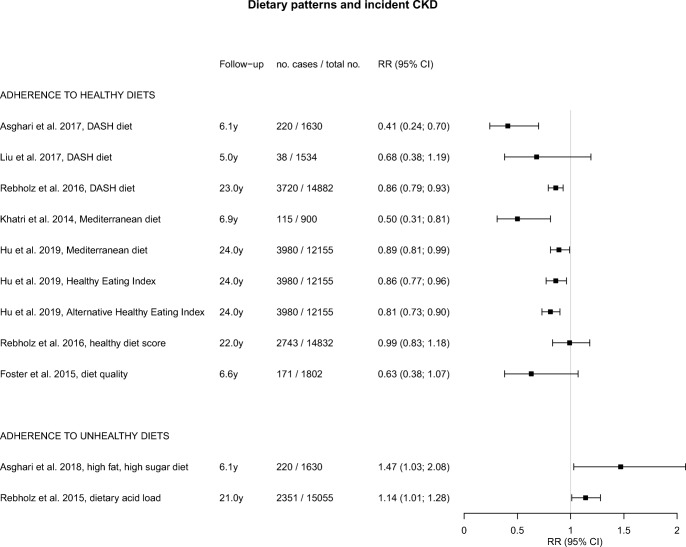
Forest plot for associations between dietary patterns and incident chronic kidney disease. DASH, Dietary Approach to Stop Hypertension

An overview of studies of foods, beverages, and dietary patterns and their associations with incident CKD is presented in eTable 1. Studies that focused on eGFR change, albuminuria, or hyperuricemia are described in eTable 2 and eTable 3. Graphical displays of the point estimates with 95% CI related to incident CKD using forest plots are presented in Figs. 1, 2, and 3.

### Foods

#### Meat

Two studies evaluated the consumption of red (processed) meat and poultry in relation to incident CKD (Fig. 1) [25••, 26••]. Red meat intake in these studies varied between 0.17 to 0.34 servings per day (low intake) and 1.15 to 2.52 servings per day (high intake). In the Atherosclerosis Risk in Communities (ARIC) study of ~ 12,000 US participants with 23 years of follow-up, a total of 2632 participants developed CKD [25••]. In this population, the HR for high versus low intake of red meat and CKD risk was 1.19 (95% CI, 1.03; 1.36; Fig. 1) [25••]. In a study of 4881 Iranian participants followed for 3 years, 613 participants developed CKD with an OR of 1.73 (95% CI, 1.33; 2.24) for high versus low red meat intake (Fig. 1) [26••]. Findings for processed meat were similar to those for red meat in both studies, and no significant associations with kidney function were found for poultry (Fig. 1) [25••].

#### Fish

Two studies evaluated the association between fish consumption and incident CKD (Fig. 1) [25••, 27]. The Strong Heart Study among American Indians followed 2261 participants for 5.4 years of whom 4% developed CKD. Fish intake was analyzed in four categories ranging from 0 to > 15 g per day [27]. No significant associations were found with an OR of 1.46 (95% CI, 0.65; 3.26) for high versus zero fish intake [27]. In the ARIC study [25••], fish intake was analyzed in quintiles ranging from 0.07 to 0.64 servings per day. A borderline significant HR of 0.89 (95% CI, 0.78; 1.01) was found in the upper versus lower quintile of intake (Fig. 1) [25••].

#### Dairy

Dairy consumption and incident CKD were examined in the ARIC study among US individuals (Fig. 1) [25••]. Intake of low-fat dairy ranged from 0.00 to 2.04 servings per day and intake of high-fat dairy from 0.13 to 1.61 servings per day [25••]. A significantly lower risk of CKD was found for low-fat dairy intake, with a HR of 0.75 (95% CI, 0.65; 0.85) for high versus low intake. High-fat dairy intake was also inversely associated with CKD, albeit non-significant (Fig. 1) [25••].

#### Vegetables

We found 3 studies of vegetable intake and CKD risk (Fig. 1) [28•, 29••, 30]. In a study of 1780 Iranians from the Tehran Lipid Glucose Study (TLGS), followed for 6 years, 319 participants developed CKD [28•]. Allium vegetable intake was analyzed in tertiles ranging from 1 to 39 g per week [28•]. A significant inverse association with CKD risk was found, with a HR of 0.68 (95% CI, 0.48; 0.98) in the upper versus lower tertiles of intake [28•]. In 9229 participants from the Korean Genome and Epidemiology Study, 1741 incident CKD cases were reported during 8.2 years [29••]. Intake of non-fermented vegetables ranged from 49 to 222 g per day, and intake of fermented vegetables from 164 to 227 g per day [29••]. Non-fermented vegetables were inversely related to CKD risk, with a HR of 0.86 (95% CI, 0.76; 0.98) for high versus low intake (Fig. 1) [29••]. For fermented vegetables, an inverse but non-significant association was found (Fig. 1) [29••]. In the abovementioned TLGS, nitrate-containing vegetable intake ranged from 146 to 428 g per day [30]. No significant association with CKD risk was found after 3 years of follow-up (Fig. 1) [30].

#### Legumes and Nuts

In the ARIC study with 23 years of follow-up, legume intake ranged from 0.07 to 0.68 servings per day and nut intake ranged from 0.03 to 0.86 servings per day [25••]. Both legumes and nuts were significantly associated with lower risks of CKD, with HRs of 0.83 (95% CI, 0.72; 0.95) and 0.81 (95% CI, 0.72; 0.92) for high versus low intakes, respectively (Fig. 1) [25••].

#### Fruits

One study in 9229 South Koreans, followed for 8.2 years, reported on fruit consumption and incident CKD [29••]. Fruit intake ranged from 143 to 345 g per day and showed no association with incident CKD (HR of 1.00) (Fig. 1) [29••].

### Beverages

#### Coffee

Three studies examined coffee consumption and incident CKD (Fig. 2) [31–33]. The Iranian TLGS compared coffee drinkers (median intake 8.3 ml per day) to non-drinkers [31]. In the ARIC study in the USA [32] and the Korean Genome and Epidemiology Study in South Korea [33], those drinking at least 3 cups [32] or at least 2 cups [33] were compared with non-coffee drinkers. In the Iranian study, a non-significant direct association between coffee and CKD was found [31], whereas in the US and Korean studies, significant inverse associations were observed in those with higher coffee intakes, with HR of 0.84 [32] and 0.80 [33], respectively (Fig. 2).

#### Tea

The Iranian TLGS also reported on tea consumption, ranging from < 250 ml (low intake) to > 750 ml per day (high intake) (Fig. 2) [31]. Unfortunately, data on the type of tea and its preparation method was not collected [31]. However, a previous study reported that in Iran, black tea is often consumed [34] with added sweets and sugar, including a variety of additives [31]. No significant association with incident CKD was found (Fig. 2) [31].

#### Soft Drinks

Three studies reported on SSBs and incident CKD (Fig. 2) [35–37], of which one American study also reported on diet beverages (Fig. 2) [36]. In the ARIC study with 9 years of follow-up, consumption of SSB (cutoff 1 drink per day) was not significantly associated with CKD risk [35]. In the Jackson Heart Study (3003 participants, 185 CKD cases) with 8 years of follow-up, a direct, non-significant association of SSB with CKD risk was found [36]. In the Iranian TLGS, SSB consumption ranged from < 0.5 to > 4 servings per week [37]. A significantly elevated risk of CKD was found when comparing high with low intakes, with an OR (95% CI) of 1.92 (1.05; 3.48) (Fig. 2) [37]. Diet beverages were studied in the Jackson Heart Study and showed no significant association with CKD risk (Fig. 2) [36].

### Dietary Patterns

#### Healthy Diets

A number of studies examined healthy dietary patterns and incident CKD [38, 39, 40•, 41, 42••, 43, 44], including the DASH diet [39, 40•, 41], Mediterranean diet [38, 42••], and other healthy dietary patterns [42••, 43, 44], for which findings are shown in Fig. 3. The DASH diet was examined in the Healthy Aging in Neighborhoods of Diversity across the Life Span cohort with 5 years of follow-up [40•], in the ARIC study with 23 years of follow-up [41] and in the Iranian TLGS with 6.1 years of follow-up [39]. All studies suggested a beneficial effect of the DASH diet, with RRs between 0.41 and 0.86 for high versus low adherence (Fig. 3). The association was statistically significant for 2 studies [39, 41].

Mediterranean diet scores were examined in the Northern Manhattan Study [38] and ARIC study [42••], with 6.9 years [38] and 24 years [42••] of follow-up, respectively. Reduced RRs of 0.50 [38] and 0.89 [42••] were found for high versus low adherence, which were significant for both studies (Fig. 3).

The ARIC study also examined [42••] adherence to healthy dietary patterns assessed using the Healthy Eating Index-2015 (HEI-2015) and the alternative HEI-2010 [42••]. The HEI-2015 was designed to assess adherence to US Dietary Guidelines for Americans [45], while the alternative HEI-2010 was designed to identify key components associated with chronic diseases [46]. For both diet quality scores, significantly lower risks of CKD were found for higher adherence, with RRs of 0.86 and 0.81, respectively (Fig. 3) [42••].

In the ARIC study with 22 years of follow-up, the Healthy Diet Score based on American Heart Association’s Life’s Simple 7 was studied, which appeared not to be associated with incident CKD [43]. In the Framingham Offspring cohort followed for 6.6 years (1802 participants, 171 CKD cases), the Dietary Guidelines Adherence Index was borderline significantly inversely associated with CKD risk [44].

Healthy dietary patterns were also beneficially associated with other renal function outcomes, such as rapid eGFR decline [40•, 44] and ≥ 25% eGFR decline [40•] (Supplementary material; eTable 1).

#### Unhealthy Diets

Two studies reported on unhealthy dietary patterns and incident CKD (Fig. 3) [47, 48]. In the TLGS, a high-fat, high-sugar diet was related to a significantly higher risk of CKD, with OR of 1.46 [47]. In participants of the ARIC study, an increased HR of 1.13 was found for a diet with a high acid load (12.2 to 100.7 mEq per day), which is characterized by high levels of salt, animal protein, and phosphorus, compared with a low acid load (− 119.1 to − 3.2 mEq per day).

## Conclusion

This review of 21 prospective cohort studies among individuals with (relatively) normal kidney function shows a consistently lower risk of CKD in those adhering to a healthy dietary pattern [38, 39, 40•, 41, 42••, 43, 44]. For individual food groups and beverages, the observed associations were more variable and weaker. We found adverse associations for red (processed) meat and SSBs in some studies and beneficial associations for dairy, vegetables, legumes, and nuts.

Two recent reviews have indicated that healthy dietary patterns may prevent incident CKD [15, 16]. Ajjarapu et al. included 26 prospective cohort studies and found that adherence to a DASH or Mediterranean diet may be useful to prevent CKD [16]. Similar results were found in a meta-analysis of 15 prospective and retrospective cohort studies performed by Bach et al. [15]. A low animal/vegetable protein ratio is often considered an indicator of a healthy dietary pattern. In this regard, the ARIC study [25••] showed that after 23 years of follow-up, high (> 22.8 g per day) versus low (< 12.1 g per day) intake of vegetable protein was significantly associated with lower risk of CKD, whereas no association was found for high (> 69.6 g per day) versus low (< 36.4 g per day) intake of animal protein [25••]. Similar results on animal protein intake were found in 1135 participants with normal renal function (defined as eGFR > 80 ml/min/1.73 m^2^) from the Nurses’ Health Study [49].

A lower risk of incident CKD for those consuming more vegetables and legumes may partly be attributable to fiber, as shown in a study among Iranian TLGS participants, with 6.1 years of follow-up [50]. Consumption of whole grains has also been linked to less kidney function decline in the Doetinchem Study in The Netherlands, with 15 years of follow-up [51]. In a study of vegetables and fruit intake in relation to kidney function decline, assessed by the annual change in eGFR, inverse associations were found [51] (Supplementary material; eTable 2), which strengthens our findings on healthy dietary patterns.

We found no association of CKD with fish intake, which is often considered part of a healthy diet. This was confirmed in another study among American Indians with 5.4 years of follow-up, where fish intake was not related to change in kidney function [27] (Supplementary material; eTable 2). For poultry, we could only include one study, and more research is needed.

Our results for coffee, indicating a potentially protective effect, are also in line with the results from a study on kidney function change [52] (Supplementary material; eTable 2). In this study, the coffee was mainly caffeinated [52] and likely to be filtered. The Iranian study suggested an increased, albeit non-significant, risk of CKD, which could be attributable to the regularly consumed unfiltered type of coffee in this country [31]. However, more information regarding the type of coffee and its preparation methods is needed, including amounts of added sugar and other condiments, before results can be correctly interpreted. We found no beneficial associations for tea and incident CKD, which was in line with the results from a Dutch study on kidney function decline [52] (Supplementary material; eTable 2). However, our review included only one study on incident CKD from Iran [31]. More information about the types of tea in relation to kidney function, including amounts of added sugar, is needed before drawing conclusions.

For low-fat dairy products and incident CKD, we found some evidence for a potentially protective effect on kidney function, though based on only one study [25••]. This is in line with a study in Dutch participants in which less kidney function loss was found during 15 years of follow-up who consumed more milk and low-fat dairy [53] (Supplementary material; eTable 2).

With regard to other kidney function outcomes (Supplementary material; eTable 3), studies on the risk of albuminuria [27, 35, 54, 55] and hyperuricemia [35] were in accordance with those for CKD. A higher, albeit non-significant risk of hyperuricemia was found for high versus low SSB consumption [35]. Also, a good versus poor diet quality, based on eight fundamental DASH diet components, was associated with a lower risk of incident microalbuminuria [55], and fruit intake was related to a lower risk of albuminuria [54]. Fish intake was not associated with albuminuria [27, 56].

To summarize, this review shows that a healthy dietary pattern may help prevent kidney function decline and lower the risk of CKD. The number of studies of individual foods and beverages in this field, however, is limited and most of the evidence comes from a limited number of cohorts. More research on the components of healthy (and unhealthy) diets and indicators of kidney health in different populations is needed to fill these knowledge gaps.

## Electronic Supplementary Material

ESM 1 (DOCX 28.9 kb)

ESM 2 (DOCX 66.7 kb)

ESM 3 (DOCX 19.3 kb)

ESM 4 (DOCX 19.5 kb)
