# Supplementary material for: Diet and Kidney Function: a Literature Review
Source: Curr Hypertens Rep. 2020 Feb 3;22(2):14. doi: 10.1007/s11906-020-1020-1 (PMC6997266; doi:10.1007/s11906-020-1020-1)
Supplement: Supplementary file 1 — (DOCX 28.9 kb) [file 11906_2020_1020_MOESM1_ESM.docx]

**1. Foods**

Number of articles retrieved:

Meat: n=816

Fish: n=1,939

Dairy: n=80

Vegetables, legumes, nuts: n=645

Fruit: n=870

Number of articles retrieved:

Meat: n=5

Fish: n=5

Dairy: n=3

Vegetables, legumes, nuts: n=6

Fruit: n=1

Remaining number of articles:

Meat: n=2

Fish: n=3

Dairy: n=1

Vegetables, legumes, nuts: n=4

Fruit: n=1

**Title/abstract**

**screening**

**Full-text**

**review**

18 full-text articles excluded for the following reasons:

Short-term follow-up: n=3

Other study design: n=2

Other outcome than incident CKD: n=8

No full-text available: n=2

Population with pre-existing condition: n=1

**2. Beverages**

Number of articles retrieved:

Coffee: n=395

Tea: n=385

Soft drinks: n=108

Number of articles retrieved:

Coffee: n=5

tea: n=3

Soft drinks: n=6

Remaining number of articles:

Coffee n=3

Tea: n=1

Soft drinks: n=3

**3. Dietary patterns**

Number of articles retrieved:

Diets: n=12,375

Number of articles retrieved:

Diets: n=16

Remaining number of articles:

Diets: n=10

**eFigure 1.** Flowchart of the literature search on diet and kidney function.
