# Supplementary material for: Diet and Kidney Function: a Literature Review
Source: Curr Hypertens Rep. 2020 Feb 3;22(2):14. doi: 10.1007/s11906-020-1020-1 (PMC6997266; doi:10.1007/s11906-020-1020-1)
Supplement: Supplementary file 2 — (DOCX 66.7 kb) [file 11906_2020_1020_MOESM2_ESM.docx]

**eTable 1.** Overview of prospective population-based studies of food and beverage intake and risk of chronic kidney diseases

| Food, beverage or dietary pattern | Author, year | Study population, country | Baseline characteristics | No. cases / total no. | Follow-up period | Dietary assessment | Outcome definition + ascertainment | Fully adjusted point estimate (95% CI)^a^ | Confounders |
| --- | --- | --- | --- | --- | --- | --- | --- | --- | --- |
| Red meat | Haring *et al.* 2017 | Community-based ARIC study, USA | ●Women: 56%  ●Age: 54 ± 6y  ●Black race: 23%  ●eGFR: 103 ± 14 ml/min/1.73m^2^  ●Animal protein intake: 46 ± 8 g/day | 2,632 / 11,952 | Median: 23.0y | Validated 66-item FFQ | ●Incident CKD, defined as meeting one of the following criteria:  1)eGFR decrease ≥25% from baseline resulting in eGFR <60 ml/min/1.73m^2^ 2)CKD-related hospitalization 3)CKD-related death or 4)ESRDs  ●eGFR calculated with 2009 CKD-EPI equation_creatinine_ | Q5 vs Q1  HR (95% CI) = 1.19 (1.03; 1.36)* | ● age, sex, race-center  ● BMI, WHR  ● current smoker  ● alcohol intake  ● PA index, leisure-related PA  ● education level  ● total caloric intake  ● total carbohydrate intake  ● HDL-c, LDL-c, TGs, total cholesterol  ● SBP  ● lipid-lowering medication use, anti-hypertensive medication use |
| Red meat | Mirmiran *et al.* 2019 | Community-based TLGS, Iran | ●Men: 54%  ●Age: 40 ± 13y  ●eGFR: 76 ± 0.2 ml/min/1.73m^2^  ●Red meat intake: 1 serving/day | 613 / 4,881 | Median: 3.1y | Validated 168-item FFQ | ●Incident CKD, defined as eGFR<60 ml/min/1.73m^2^ ●eGFR assessed with MDRD equation | Q4 vs Q1  OR (95% CI) =  1.73 (1.33; 2.24)* | ● age, sex  ● BMI  ● smoking  ● PA  ● total energy intake  ● prevalent diabetes  ● TGs  ● hypertension |
| Processed meat | Haring *et al.* 2017 | Community-based ARIC study, USA | ●Women: 56%  ●Age: 54 ± 6y  ●Black race: 23%  ●eGFR: 103 ± 14 ml/min/1.73m^2^  ●Animal protein intake: 46 ± 8 g/day | 2,632 / 11,952 | Median: 23.0y | Validated 66-item FFQ | ●Incident CKD, defined as meeting one of the following criteria:  1)eGFR decrease ≥25% from baseline resulting in eGFR <60 ml/min/1.73m^2^ 2)CKD-related hospitalization 3)CKD-related death or 4)ESRDs  ●eGFR calculated with 2009 CKD-EPI equation_creatinine_ | Q5 vs Q1  HR (95% CI) = 1.12 (0.98; 1.29) | ● age, sex, race-center  ● BMI, WHR  ● current smoker  ● alcohol intake  ● PA index, leisure-related PA  ● education level  ● total caloric intake  ● total carbohydrate intake  ● HDL-c, LDL-c, TGs, total cholesterol  ● SBP  ● lipid-lowering medication use, anti-hypertensive medication use |
| Processed red meat | Mirmiran *et al.* 2019 | Community-based TLGS, Iran | ●Men: 54%  ●Age: 40 ± 13y  ●eGFR: 76 ± 0.2 ml/min/1.73m^2^  ●Red meat intake: 1 serving/day | 613 / 4,881 | Median: 3.1y | Validated 168-item FFQ | ●Incident CKD, defined as eGFR<60 ml/min/1.73m^2^ ●eGFR assessed with MDRD equation | Q4 vs Q1  OR (95% CI) =  1.99 (1.54; 2.56)* | ● age, sex  ● BMI  ● smoking  ● PA  ● total energy intake  ● prevalent diabetes  ● TGs  ● hypertension |
| Poultry | Haring *et al.* 2017 | Community-based ARIC study, USA | ●Women: 56%  ●Age: 54 ± 6y  ●Black race: 23%  ●eGFR: 103 ± 14 ml/min/1.73m^2^  ●Animal protein intake: 46 ± 8 g/day | 2,632 / 11,952 | Median: 23.0y | Validated 66-item FFQ | ●Incident CKD, defined as meeting one of the following criteria:  1)eGFR decrease ≥25% from baseline resulting in eGFR <60 ml/min/1.73m^2^ 2)CKD-related hospitalization 3)CKD-related death or 4)ESRDs  ●eGFR calculated with 2009 CKD-EPI equation_creatinine_ | Q5 vs Q1  HR (95% CI) = 0.94 (0.84; 1.06) | ● age, sex, race-center  ● BMI, WHR  ● current smoker  ● alcohol intake  ● PA index, leisure-related PA  ● education level  ● total caloric intake  ● total carbohydrate intake  ● HDL-c, LDL-c, TGs, total cholesterol  ● SBP  ● lipid-lowering medication use, anti-hypertensive medication use |
| Fish | Lee *et al.* 2012 | Strong Heart Study in American Indians, USA | ●Men: 38%  ●Age: 38 ± 16y  ●eGFR: 102 ± 26 ml/min/1.73m^2^  ●Fish intake  0 g/day: 18%  >15.0 g/day: 13%  ≤15 g/day: 69% | Unknown / 2,261 | Mean: 5.4y | 119-item Block FFQ | ●Incident CKD, defined as eGFR<60 ml/min/1.73m^2^ ●eGFR assessed with MDRD equation | >15 g/day vs 0 g/day  OR (95% CI) =  1.46 (0.65; 3.26) | ● age, sex, center  ● WHR  ● smoking  ● total energy intake  ● protein intake, sodium intake  ● prevalent diabetes  ● TGs  ● SBP  ● urinary ACR |
| Fish and seafood | Haring *et al.* 2017 | Community-based ARIC study, USA | ●Women: 56%  ●Age: 54 ± 6y  ●Black race: 23%  ●eGFR: 103 ± 14 ml/min/1.73m^2^  ●Animal protein intake: 46 ± 8 g/day | 2,632 / 11,952 | Median: 23.0y | Validated 66-item FFQ | ●Incident CKD, defined as meeting one of the following criteria:  1)eGFR decrease ≥25% from baseline resulting in eGFR <60 ml/min/1.73m^2^ 2)CKD-related hospitalization 3)CKD-related death or 4)ESRDs  ●eGFR calculated with 2009 CKD-EPI equation_creatinine_ | Q5 vs Q1  HR (95% CI) = 0.89 (0.78; 1.01) | ● age, sex, race-center  ● BMI, WHR  ● current smoker  ● alcohol intake  ● PA index, leisure-related PA  ● education level  ● total caloric intake  ● total carbohydrate intake  ● HDL-c, LDL-c, TGs, total cholesterol  ● SBP  ● lipid-lowering medication use, anti-hypertensive medication use |
| Low-fat dairy and high-fat | Haring *et al.* 2017 | Community-based ARIC study, USA | ●Women: 56%  ●Age: 54 ± 6y  ●Black race: 23%  ●eGFR: 103 ± 14 ml/min/1.73m^2^  ●Animal protein intake: 46 ± 8 g/day | 2,632 / 11,952 | Median: 23.0y | Validated 66-item FFQ | ●Incident CKD, defined as meeting one of the following criteria:  1)eGFR decrease ≥25% from baseline resulting in eGFR <60 ml/min/1.73m^2^ 2)CKD-related hospitalization 3)CKD-related death or 4)ESRDs  ●eGFR calculated with 2009 CKD-EPI equation_creatinine_ | **Low-fat dairy**  Q5 vs Q1  HR (95% CI) = 0.75 (0.65; 0.85)*  **High-fat dairy**  Q5 vs Q1  HR (95% CI) = 0.93 (0.81; 1.06) | ● age, sex, race-center  ● BMI, WHR  ● current smoker  ● alcohol intake  ● PA index, leisure-related PA  ● education level  ● total caloric intake  ● total carbohydrate intake  ● HDL-c, LDL-c, TGs, total cholesterol  ● SBP  ● lipid-lowering medication use, anti-hypertensive medication use |
| Allium vegetables | Bahadoran *et al.* 2017 | Community-based TLGS, Iran | ●Men: 44%  ●Age: 40 ± 14y  ●eGFR: 78 ± 0.3 ml/min/1.73m^2^  ●Allium vegetables intake: 17 g/week | 319 / 1,780 | Mean: 6.0y | Validated 168-item semiquantitative FFQ | ●Incident CKD defined as eGFR<60 ml/min/1.73m^2^  ●eGFR assessed with CKD-EPI_creatinine_ equation | T3 vs T1  HR (95% CI) = 0.68 (0.46; 0.98)* | ● age, sex  ● BMI  ● smoking  ● PA  ● dietary pattern scores  ● T2D  ● TGs to HDL-c ratio |
| Vegetables | Jhee *et al.* 2019 | Community-based KoGES, South Korea | ●Men: 48%  ●Age: 52 ± 9y  ●eGFR: 94 ± 14 ml/min/1.73m^2^  ●Non-fermented vegetable intake: 129 ± 127 g/day  ●Fermented vegetable intake: 203 ± 145 g/day | 1,741 / 9,229 | Mean: 8.2y | Validated 106-item semiquantitative FFQ | ●Incident CKD defined as eGFR<60 ml/min/1.73m^2^ ● eGFR assessed with CKD-EPI_creatinine_ equation | **Nonfermented vegetables**  T3 vs T1  HR (95% CI) = 0.86 (0.76; 0.98)*  **Fermented vegetables**  T3 vs T1  HR (95% CI) = 0.94 (0.83; 1.06) | ● age, sex  ● BMI  ● smoking status  ● alcohol status  ● PA  ● education level  ● total energy intake  ● red or nonred meat, fish, dairy, egg, legume, nut, grain intake  ● history of hypertension and diabetes  ● LDL-c  ● SBP  ● serum albumin, hemoglobin  ● eGFR, proteinuria level |
| NCVs | Mirmiran *et al.* 2016 | Community-based TLGS, Iran | ●Men: 43%  ●Age: 38 ± 12y  ●eGFR: 80 ± 1 ml/min/1.73m^2^  ●NCVs intake: 298 ± 177 g/day | Unknown / 1,299 | Mean: 3.0y | Validated 168-item semiquantitative FFQ | ●Incident CKD, defined as eGFR<60 ml/min/1.73m^2^ ●eGFR assessed with CKD-EPI_creatinine_ equation | T3 vs T1  OR (95% CI) =  0.93 (0.43; 2.02) | ● age, sex  ● BMI  ● smoking  ● PA  ● education  ● dietary intake of energy  ● fiber, potassium intake  ● diabetes  ● hypertension |
| Legumes | Haring *et al.* 2017 | Community-based ARIC study, USA | ●Women: 56%  ●Age: 54 ± 6y  ●Black race: 23%  ●eGFR: 103 ± 14 ml/min/1.73m^2^  ●Animal protein intake: 46 ± 8 g/day | 2,632 / 11,952 | Median: 23.0y | Validated 66-item FFQ | ●Incident CKD, defined as meeting one of the following criteria:  1)eGFR decrease ≥25% from baseline resulting in eGFR <60 ml/min/1.73m^2^ 2)CKD-related hospitalization 3)CKD-related death or 4)ESRDs  ●eGFR calculated with 2009 CKD-EPI equation_creatinine_ | Q5 vs Q1  HR (95% CI) = 0.83 (0.72; 0.95)* | ● age, sex, race-center  ● BMI, WHR  ● current smoker  ● alcohol intake  ● PA index, leisure-related PA  ● education level  ● total caloric intake  ● total carbohydrate intake  ● HDL-c, LDL-c, TGs, total cholesterol  ● SBP  ● lipid-lowering medication use, anti-hypertensive medication use |
| Nuts | Haring *et al.* 2017 | Community-based ARIC study, USA | ●Women: 56%  ●Age: 54 ± 6y  ●Black race: 23%  ●eGFR: 103 ± 14 ml/min/1.73m^2^  ●Animal protein intake: 46 ± 8 g/day | 2,632 / 11,952 | Median: 23.0y | Validated 66-item FFQ | ●Incident CKD, defined as meeting one of the following criteria:  1)eGFR decrease ≥25% from baseline resulting in eGFR <60 ml/min/1.73m^2^ 2)CKD-related hospitalization 3)CKD-related death or 4)ESRDs  ●eGFR calculated with 2009 CKD-EPI equation_creatinine_ | Q5 vs Q1  HR (95% CI) = 0.81 (0.72; 0.92)* | ● age, sex, race-center  ● BMI, WHR  ● current smoker  ● alcohol intake  ● PA index, leisure-related PA  ● education level  ● total caloric intake  ● total carbohydrate intake  ● HDL-c, LDL-c, TGs, total cholesterol  ● SBP  ● lipid-lowering medication use, anti-hypertensive medication use |
| Fruit | Jhee *et al.* 2019 | Community-based KoGES, South Korea | ●Men: 48%  ●Age: 52 ± 9y  ●eGFR: 94 ± 14 ml/min/1.73m^2^  ●fruit intake: 269 ± 332 g/day | 1,741 / 9,229 | Mean: 8.2y | Validated 106-item semiquantitative FFQ | ●Incident CKD defined as eGFR <60 ml/min/1.73m^2^ ●eGFR assessed with CKD-EPI_creatinine_ | T3 vs T1  HR (95% CI) = 1.00 (0.88; 1.14) | ● age, sex  ● BMI  ● smoking status  ● alcohol status  ● PA  ● education level  ● total energy intake  ● red or nonred meat, fish, dairy, egg, legume, nut, grain intake  ● history of hypertension and diabetes  ● LDL-c  ● SBP  ● serum albumin, hemoglobin  ● eGFR, proteinuria level |
| Coffee | Gaeini *et al.* 2019 | Community-based TLGS, Iran | ●Men: 41%  ●Age: 34 ± 15y  ●eGFR: 79 ± 12 ml/min/1.73m^2^  ●Coffee intake: 14 ± 55 ml/day | 318 / 1,780 | Mean: 6.4y | Validated 168-item FFQ | ●Incident CKD, defined as eGFR <60 ml/min/1.73m^2^ ●eGFR assessed with CKD-EPI_creatinine_ equation | Drinker vs non-drinker:  HR (95% CI) = 1.17 (0.90; 1.51) | ● age, sex  ● BMI  ● smoking  ● total energy  ● fiber, tea  ● dietary fat  ● TGs to HDL-c ratio |
| Coffee | Hu *et al.* 2018 | Community-based ARIC Study, USA | ●Women: 56%  ●Age: 54 ± 6y  ●White race: 75%  ●Black race: 25%  ●eGFR: 103 ± 14 ml/min/1.73m^2^  ●Coffee intake  Never: 19%  <1 cup/d: 21%  ≥1-<2 cups/d: 25%  ≥2-<3 cups/d: 15%  ≥3 cups/d:19% | 3,845 / 14,209 | Median: 24.0y | 66-item semi-quantitative FFQ | ●Incident CKD defined as meeting at least one of following criteria:  1)eGFR<60ml/min/1.73m^2^  2)ICD-9/10 code for CKD stage ≥3 related hospitalization  3)ICD-9/10 code for death related to CKD stage ≥3  4)ESRD  ●eGFR assessed with CKD-EPI_creatinine_ equation | ≥3 cups/d vs non-coffee drinker  HR (95% CI) = 0.84 (0.75; 0.94)* | ● age, sex, race-center  ● BMI  ● smoking  ● alcohol status  ● PA  ● education  ● total energy intake  ● DASH diet score  ● diabetes status  ● SBP  ● anti-hypertensive medication use  ● baseline eGFR |
| Coffee | Jhee *et al.* 2018 | Community-based KoGES, South Korea | ●Men: 48%  ●Age: 52 ± 9y  ●eGFR: 94 ± 14 ml/min/1.73m^2^  ●Coffee intake  Never: 23.0%  <1 cup/week: 7%  1-6 cups/week: 18%  1 cup/day: 27%  ≥2 cups/day: 26% | 828 / 8,717 | Mean: 11.3y | Validated semi-quantitative FFQ | ●Incident CKD defined as eGFR<60 ml/min/1.73m^2^  ●eGFR assessed with CKD-EPI_creatinine_ | ≥2 cups/d vs non-coffee drinker  HR (95% CI) = 0.80 (0.65; 0.98)* | ● age, sex  ● BMI  ● smoking status  ● alcohol status  ● education levels, income  ● daily intake amount of tea and chocolate  ● history of hypertension and CVD events  ● HbA1c, history of diabetes  ● CRP, hemoglobin, albumin  ● total cholesterol  ● mean arterial pressure  ● eGFR, proteinuria |
| Tea | Gaeini *et al.* 2019 | Community-based TLGS, Iran | ●Men: 41%  ●Age: 34 ± 15y  ●eGFR: 79 ± 12 ml/min/1.73m^2^  ●Tea intake: 570 ± 553 ml/day | 318 / 1,780 | Mean: 6.4y | Validated 168-item FFQ | ●Incident CKD, defined as eGFR<60 ml/min/1.73m^2^ ●eGFR assessed with CKD-EPI_creatinine_ equation | >750 ml/d vs <250 ml/d:  HR (95% CI) = 0.92 (0.68; 1.25) | ● age, sex  ● BMI  ● smoking  ● total energy  ● fiber, coffee  ● dietary fat  ● TGs to HDL-c ratio |
| Sugar-sweetened soda | Bomback *et al.* 2010 | Community-based ARIC Study, USA | ●Women: 55%  ●Age: 54 ± 6y  ●Race  White: 73%  Black: 27%  Other: 0.3%  ●eGFR: 92 ± 21 ml/min/1.73m^2^  ●Soda drinking  <1 soda/day: 82%  1 soda/day: 12%  >1 soda/day: 6% | 1,160 / 14,002 | Mean: 9.0y | Validated 66-item semiquantitative FFQ | ●Incident CKD defined as eGFR <60 ml/min/1.73m^2^ ●eGFR assessed with MDRD equation | >1 soda/d vs <1 soda/d  OR (95% CI) =  0.82 (0.59; 1.16) | ● age, sex, race, ARIC-field center  ● BMI  ● current tobacco use  ● alcohol use  ● caloric intake  ● sodium intake  ● diabetes  ● hypertension |
| SSB | Rebholz *et al.* 2019 | Community-based Jackson Heart Study, USA | ●Women: 64%  ●Age: 54 ± 12y  ●eGFR: 98 ± 18 ml/min/1.73m^2^ | 185 / 3,003 | Median: 8.0y | validated 158-item modified, version of Lower Mississippi Delta Nutrition Intervention Research Initiative FFQ | ●Incident CKD defined as eGFR <60 ml/min/1.73m^2^ accompanied by ≥30% eGFR decline  ●eGFR assessed with CKD-EPI_creatinine_ equation | T3 vs T1  OR (95% CI) = 1.37 (0.86; 2.16) | ● age, sex  ● BMI  ● smoking status  ● PA index  ● education  ● total energy intake  ● healthy and Southern dietary pattern score  ● diabetes  ● history of CVD  ● HDL-c, LDL-c  ● hypertension  ● baseline eGFR |
| SSBs and SSSDs | Yuzbashian *et al.* 2016 | Community-based TLGS, Iran | ●Women: 54%  ●Age: 45 ± 12y  ●eGFR: 70 ± 15 ml/min/1.73m^2^  ●SSB intake: 2 servings/week | 172 / 1,690 | Mean: 3.0y | 168-item FFQ | ●Incident CKD defined as eGFR <60 ml/min/1.73m^2^  ●eGFR assessed with MDRD equation | **SSBs**  >4 servings/week vs <0.5 servings/week  OR (95% CI) =  1.92 (1.05; 3.48)*  **SSSDs**  >4 servings/week vs <0.5 servings/week  OR (95% CI) =  2.04 (1.06; 3.91)* | ● age, sex  ● BMI  ● smoking  ● PA  ● energy intake  ● sodium  ● diabetes  ● hypertension |
| Diet beverages | Rebholz *et al.* 2019 | Community-based Jackson Heart Study, USA | ●Women: 64%  ●Age: 54 ± 12y  ●eGFR: 98 ± 18 ml/min/1.73m^2^ | 185 / 3,003 | Median: 8.0y | Validated 158-item modified, version of Lower Mississippi Delta Nutrition Intervention Research Initiative FFQ | ●Incident CKD defined as eGFR <60 ml/min/1.73m^2^ accompanied by ≥30% eGFR decline  ●eGFR assessed with CKD-EPI_creatinine_ equation | T3 vs T1  OR (95% CI) = 0.80 (0.51; 1.25) | ● age, sex  ● BMI  ● smoking status  ● PA index  ● education  ● total energy intake  ● healthy and Southern dietary pattern score  ● diabetes  ● history of CVD  ● HDL-c, LDL-c  ● hypertension  ● baseline eGFR |
| Adherence to DASH diet | Asghari *et al.* 2017 | Community-based TLGS, Iran | ●Women: 51%  ●Age: 43 ± 11y  ●eGFR: 73 ± 8 ml/min/1.73m^2^●DASH diet score: 24 | 220 / 1,630 | Median: 6.1y | Validated 168-item FFQ | ●Incident CKD, defined as eGFR <60 ml/min/1.73m^2^  ●eGFR assessed with MDRD equation | Q5 vs Q1  OR (95% CI) =  0.41 (0.24; 0.70)* | ● age, sex  ● BMI  ● smoking  ● PA  ● total energy intake  ● diabetes  ● TGs  ● hypertension  ● eGFR |
| Adherence to DASH diet | Liu *et al.* 2017 | Population-based HANDLS study, USA | ●Men: 42%  ●Age: 48 ± 9y  ●African American race: 59%  ●eGFR: 95 [82; 108] ml/min/1.73m^2^  ●DASH accordance score: 2 [1; 3] | **Incident CKD**  38 / 1,534  **Rapid eGFR decline**  193 / 1,534  **eGFR decline ≥25% during follow-up**  65 / 1,534 | Median: 5.0y | 24h self-reported food intake | ●Incident CKD, defined as eGFR<60 ml/min/1.73m^2^  ●Rapid eGFR decline, defined as eGFR decline >3 ml/min/1.73m^2^ per year  ●eGFR decline ≥25% from baseline onwards  ●eGFR assessed with CKD-EPI_creatinine_ equation | **Incident CKD**  High vs low DASH accordance  OR (95% CI) =  0.68 (0.38; 1.19)  **Rapid eGFR decline**  High vs low DASH accordance  OR (95% CI) =  0.82 (0.61; 1.09)  **eGFR decline ≥25%**  High vs low DASH accordance  OR (95% CI) =  0.77 (0.45; 1.32) | **Rapid kidney function decline**  ● age, sex, race  ● tobacco use  ● education level, poverty status  ● total energy intake  ● diabetes  ● hypertension, SBP  **Incident CKD +** **eGFR decline ≥25%**  ● age, sex, race  ● poverty status |
| Adherence to DASH diet | Rebholz *et al.* 2016 | Community-based ARIC study, USA | **T1 (low DASH diet score)**  ●Women: 44%  ●Age: 54 ± 6y  ●eGFR: 104 ± 15 ml/min/1.73m^2^  **T2 (moderate DASH diet score)**  ●Women: 56%  ●Age: 54 ± 6y  ●eGFR: 103 ± 14 ml/min/1.73m^2^  **T3 (high DASH diet score)**  ●Women: 68%  ●Age: 55 ± 6y  ●eGFR: 102 ± 13 ml/min/1.73m^2^ | 3,720 / 14,882 | Median: 23.0y | Semi-quantitative 66-item FFQ | ●Incident CKD, defined as  1) eGFR <60 ml/min/1.73m^2^ accompanied by ≥25% eGFR decline from baseline or 2)kidney disease related hospitalization or death or 3)ESRD  ●eGFR was calculated with 2009 CKD-EPI_creatinine_ equation | T3 vs T1  HR (95% CI)=  0.86 (0.79; 0.93)* | ● age, sex, race-center  ● overweight, obesity status  ● smoking status  ● PA  ● education level  ● total caloric intake  ● diabetes  ● hypertension, SBP  ● ACE inhibitor use, ARB use  ● baseline eGFR |
| Adherence to MeDi | Khatri *et al.* 2014 | Community-based NOMAS, USA | ●Men: 41%  ●Age: 64 ± 8y  ●eGFR: 83 ± 20 ml/min/1.73m^2^  ●Race/ethnicity  White: 15%  Black: 18%  Hispanic: 65%  Other: 3% | 115 / 900 | Mean: 6.9y | Modified Block National Cancer Institute FFQ | ●Incident CKD, defined as <60 ml/min/1.73m^2^. ●eGFR assessed with MDRD equation | **MeDi score ≥5 vs <5**  OR (95% CI) =  0.50 (0.31; 0.81)* | ● age, sex, race  ● BMI  ● smoking status  ● PA  ● education, insurance status  ● diabetes  ● LDL-c, HDL-c  ● hypertension  ● ACE inhibitor or ARB usage  ● baseline eGFR |
| Adherence to healthy dietary patterns measured by HEI-2015, aHEI-2010, alternate MeDi | Hu *et al.* 2019 | Community-based ARIC study, USA | ●Women: 69%  ●Age: 54 ± 6y  ●Black race: 23%  ●eGFR: 103 ± 14 ml/min/1.73m^2^ | 3,980 / 12,155 | Median: 24.0y | Semiquantitative 66-item FFQ | ●Incident CKD, defined as  1) eGFR <60 ml/min/1.73m^2^ accompanied by ≥25% eGFR decline from baseline or 2)kidney disease related hospitalization or death or 3)ESRD  ●eGFR assessed with CKD-EPI_creatinine_ equation | **HEI-2015**  Q5 vs Q1  HR (95% CI) = 0.86 (0.77; 0.96)*  **aHEI-2010**  Q5 vs Q1  HR (95% CI) =  0.81 (0.73; 0.90)*  **aMed**  Q5 vs Q1  HR (95% CI) =  0.89 (0.81; 0.99)* | ● age, sex, race-center  ● BMI  ● smoking status, pack-years  ● PA  ● education, income  ● total energy intake  ● dietary acid load  ● diabetes  ● HDL-c  ● SBP  ● anti-hypertensive medication use  ● eGFR |
| Healthy diet score | Rebholz *et al.* 2016 | Community-based ARIC study, USA | ●Women: 55%  ●Age: 54 ± 6y  ●Black race: 26%  ●African-American race: 26%  ●eGFR: 103 ± 14 ml/min/1.73m^2^ | 2,743 / 14,832 | Median: 22.0y | Semiquantitative 66-item FFQ | ●Incident CKD, defined as  1) eGFR <60 ml/min/1.73m^2^ accompanied by ≥25% eGFR decline from baseline or 2)kidney disease related hospitalization or death or 3)ESRD  ●eGFR was calculated with 2009 CKD-EPI_creatinine_ equation | **Ideal vs poor**  HR (95% CI) = 0.99 (0.83; 1.18) | ● age, sex, race  ● baseline eGFR |
| Diet quality | Foster *et al.* 2015 | Framingham Offspring Cohort, USA | ●Women: 55%  ●Age: 59 ± 9y  ●DGAI  Low: 6 ± 1  High: 13 ± 1 | **Incident CKD**  171 / 1,802  **Rapid eGFR decline**  238 / 1,964 | Mean: 6.6y | Harvard semi-quantitative FFQ | ●Incident CKD, defined as eGFR<60 ml/min/1.73m^2^. ●Rapid eGFR decline, defined as eGFR decline ≥3 mL/min/1.73m^2^ per year  ●eGFR assessed with CKD-EPI_creatinine_ equation | **Incident CKD**  Q4 vs Q1  OR (95% CI) =  0.63 (0.38; 1.07)  **Rapid eGFR decline**  Q4 vs Q1  OR (95% CI) =  0.69 (0.45; 1.05) | ● age, sex  ● BMI  ● diabetes  ● hypertension  ● baseline eGFR, dipstick proteinuria |
| High-fat, high-sugar diet | Asghari *et al.* 2018 | Community-based TLGS, Iran | ●Women: 51%  ●Age: 43 ± 11y  ●eGFR: 74 ± 9 ml/min/1.73m^2^ | 220 / 1,630 | Median: 6.1y | Validated 168-item FFQ | ●Incident CKD, defined as eGFR<60 ml/min/1.73m^2^.  ●eGFR assessed with MDRD equation | T3 vs T1  OR (95% CI) =  1.46 (1.03; 2.09)* | ● age, sex  ● BMI  ● smoking  ● PA  ● total energy intake  ● diabetes  ● hypertension |
| Dietary acid load | Rebholz *et al.* 2015 | Community-based ARIC study, USA | ●Women: 55%  ●Age: 54 ± 6y  ●Black: 26%  ●African-American: 26%  ●eGFR: 103 ± 14 ml/min/1.73m^2^  ●Renal acid load: 5 [-3; 12] mEq/day | 2,351 / 15,055 | Median: 21.0y | Semiquantitative 66-item FFQ | ●Incident CKD, defined as  1) eGFR <60 ml/min/1.73m^2^ accompanied by ≥25% eGFR decline from baseline or 2)kidney disease related hospitalization or death or 3)ESRD  ●eGFR assessed with CKD-EPI_creatinine_ equation | Q4 vs Q1  HR (95% CI) = 1.13 (1.01; 1.28)* | ● age, sex, race-center  ● overweight, obesity status  ● smoking  ● PA  ● education  ● total caloric intake  ● diabetes  ● hypertension  ● baseline eGFR |

^a^ Fully adjusted point estimates, but without potential mediators of the association, are presented. *Indicating statistically significant estimates. HR = hazard ratio; OR = odds ratio; CI = confidence interval; ARIC = Atherosclerosis Risk in Communities; USA = United States of America; eGFR = estimated glomerular filtration Rate; FFQ = food frequency questionnaire; CKD = chronic kidney disease; ESRD = end-stage renal disease; CKD-EPI = Chronic Kidney Disease Epidemiology collaboration; PA = physical activity; HDL = high-density lipoprotein; BMI = body mass index; WHR = waist-hip ratio; TLGS = Tehran Lipid Glucose Study; MDRD = Modification in Diet and Renal Disease; ACR = albumin-to-creatinine; TGs to HDL-c ratio = triglycerides to high-density lipoprotein-cholesterol ratio; KoGES = Korean Genome and Epidemiology Study; LDL-c = low-density lipoprotein-cholesterol; NCVs = nitrate-containing vegetables; ICD = international classification of disease; DASH = Dietary Approach to Stop Hypertension; SBP = systolic blood pressure; CVD = cardiovascular disease; HbA1c = hemoglobin A1c; CRP = C-reactive protein; SSSDs = sugar-sweetened carbonated soft drinks; SSBs = sugar-sweetened beverages; HANDLS = Healthy Aging in Neighborhoods of diversity across the Life Span; MeDi = Mediterranean diet; NOMAS = Northern Manhattan Study; ACE inhibitor = angiotensin-converting enzyme; ARB usage = angiotensin-receptor blockers; HEI = Healthy Eating Index; aHEI = alternative Healthy Eating Index; DGAI = Dietary Guidelines Adherence Index.
