# Supplementary material for: Diet and Kidney Function: a Literature Review
Source: Curr Hypertens Rep. 2020 Feb 3;22(2):14. doi: 10.1007/s11906-020-1020-1 (PMC6997266; doi:10.1007/s11906-020-1020-1)
Supplement: Supplementary file 3 — (DOCX 19.3 kb) [file 11906_2020_1020_MOESM3_ESM.docx]

**eTable 2** Overview of prospective population-based studies of food and beverage intake and its association with annual change in kidney function

| Food, beverage or dietary pattern | Author, year | Study population, country | Baseline characteristics | Total no. | Follow-up period | Dietary assessment | eGFR assessment | Fully adjusted point estimate (95% CI) | Confounders |
| --- | --- | --- | --- | --- | --- | --- | --- | --- | --- |
| Fish | Lee *et al.* 2012 | Strong Heart Study in American Indians, USA | ●Men: 38%  ●Age: 38 ± 16y  ●eGFR: 102 ± 26 ml/min/1.73m^2^  ●Fish intake  0 g/day: 18%  >15.0 g/day: 13%  ≤15 g/day: 69% | Unknown / 2,261 | Mean: 5.4y | 119-item Block FFQ | MDRD equation | >15 g/day vs 0 g/day  Beta (95% CI) = -0.34 (-0.85; 0.18) | ● age, sex, center  ● WHR  ● smoking  ● total energy intake  ● protein intake, sodium intake  ● prevalent diabetes  ● TGs  ● SBP  ● urinary ACR |
| milk(products)  Low-fat dairy | Herber-Gast *et al.* 2016 | Population-based Doetinchem cohort study, the Netherlands | ●Women: 52%  ●Age: 45 ± 10y  ●eGFR: 109 ± 14 ml/min/1.73m^2^  ●Dairy protein intake: 25 ± 10 g/day | 3,798 | Mean: 15.0y | Validated 178-item semiquantitative FFQ | CKD-EPI_cystatin-C_  equation | **Milk(products)**  T3 vs T1  Beta (95% CI)=  0.09 (0.002; 0.18)*  **Low-fat dairy**  T3 vs T1  Beta (95% CI)=  0.11 (0.02; 0.20)* | ● age, sex  ● BMI  ● smoking  ● alcohol  ● PA  ● highest attained level of education  ● daily energy intake  ● diabetes  ● hypercholesterolemia  ● hypertension |
| Vegetables | Herber-Gast *et al.* 2017 | Population-based Doetinchem cohort study, the Netherlands | ●Women: 52%  ●Age: 45 ± 10y  eGFR: 105 ± 14 ml/min/1.73m^2^  ●annual eGFR change: -0.95 ± 0.7 ml/min/1.73m^2^  ●Intake vegetables: 114 [51] g/day | 3,787 | Mean: 15.0y | Validated 178-item semiquantitative FFQ | CKD-EPI_creatinine-cystatin C_ equation | Q4 vs Q1  Beta (95% CI) = -0.04 (-0.08; 0.07) | ● age, sex  ● BMI  ● smoking  ● alcohol use  ● time-dependent PA  ● education  ● daily energy intake  ● energy-adjusted intake of total protein, low-fat dairy products, coffee and nuts, supplement use  ● diabetes  ● hypercholesterolaemia  ● hypertension |
| Fruit | Herber-Gast *et al.* 2017 | Population-based Doetinchem cohort study, the Netherlands | ●Women: 52%  ●Age: 45 ± 10y  eGFR: 105 ± 14 ml/min/1.73m^2^  ●annual eGFR change: -0.95 ± 0.7 ml/min/1.73m^2^  ●Intake fruit: 150 [153] g/day | 3,787 | Mean: 15.0y | Validated 178-item semiquantitative FFQ | CKD-EPI_creatinine-cystatin C_ equation | Q4 vs Q1  Beta (95% CI) = 0.04 (-0.03; 0.11) | ● age, sex  ● BMI  ● smoking  ● alcohol use  ● time-dependent PA  ● education  ● daily energy intake  ● energy-adjusted intake of total protein, low-fat dairy products, coffee and nuts, supplement use  ● diabetes  ● hypercholesterolaemia  ● hypertension |
| Coffee | Herber-Gast *et al.* 2016 | Population-based Doetinchem cohort study, the Netherlands | ●Men: 48%  ●Age: 46 ± 10y  eGFR: 108 ± 15 ml/min/1.73m^2^  Annual eGFR ●change: -1 ± 0.8 ml/min/1.73m^2^ | 3,786 | Mean: 15.0y | Validated 178-item FFQ | CKD-EPI_creatinine-cystatine C_  equation | >6 cups/d vs <1 cups/d  Beta (95% CI) = 0.02 (-0.09; 0.14) | ● age, sex  ● BMI  ● smoking  ● alcohol use  ● PA  ● education  ● daily energy intake  ● energy-adjusted intake of fiber, vitamin C, total protein, fat, saturated fat, tea  ● diabetes  ● hypercholesterolemia  ● hypertension |
| Tea | Herber-Gast *et al.* 2016 | Population-based Doetinchem cohort study, the Netherlands | ●Men: 48%  ●Age: 46 ± 10y  ●eGFR: 108 ± 15 ml/min/1.73m^2^  ●Annual eGFR change: -1 ± 0.8 ml/min/1.73m^2^ | 3,786 | Mean: 15.0y | Validated 178-item FFQ | CKD-EPI_creatinine-cystatine C_  equation | >4 vs <1 cups/d  Beta (95% CI) = 0.02 (-0.08; 0.11) | ● age, sex  ● BMI  ● smoking  ● alcohol use  ● PA  ● education  ● daily energy intake  ● energy-adjusted intake of fiber, vitamin C, total protein, fat, saturated fat, coffee  ● diabetes  ● hypercholesterolemia  ● hypertension |

*Indicates statistical significant. eGFR = estimated glomerular filtration rate; FFQ = food frequency questionnaire; CKD-EPI = Chronic Kidney Disease Epidemiology collaboration; BMI = body mass index; PA = physical activity.
