# Supplementary material for: Diet and Kidney Function: a Literature Review
Source: Curr Hypertens Rep. 2020 Feb 3;22(2):14. doi: 10.1007/s11906-020-1020-1 (PMC6997266; doi:10.1007/s11906-020-1020-1)
Supplement: Supplementary file 4 — (DOCX 19.5 kb) [file 11906_2020_1020_MOESM4_ESM.docx]

**eTable 3** Overview of prospective population-based studies of food and beverage intake and risk of (micro/macro)albuminuria, hyperuricemia

| Food, beverage or dietary pattern | Author, year | Study population, country | Baseline characteristics | No. cases / total no. | Follow-up period | Dietary assessment | Outcome definition + ascertainment | Fully adjusted point estimate (95% CI) | Confounders |
| --- | --- | --- | --- | --- | --- | --- | --- | --- | --- |
| Fish | Lee *et al.* 2012 | Strong Heart Study in American Indians, USA | ●Men: 38%  ●Age: 38 ± 16y  ●Fish intake  0 g/day: 18%  >15.0 g/day: 13%  ≤15 g/day: 69%  ●Albuminuria  microalbuminuria: 13%  macroalbuminuria: 3% | Unknown / 2,261 | Mean: 5.4y | 119-item Block FFQ | ●Nephropathy defined as presence of microalbuminuria (urinary ACR=30-299 mg/g) or macroalbuminuria (urinary ACR ≥300 mg/g) | **Albuminuria**  >15 g/day vs 0 g/day  OR (95% CI) = 1.21 (0.77; 1.92)  **Change in urinary ACR**  >15 g/day vs 0 g/day  Beta (95% CI) = 46.6 (-43.6; 136.9) | ● age, sex, center  ● WHR  ● smoking  ● total energy intake  ● protein intake, sodium intake  ● prevalent diabetes  ● TGs  ● SBP  ● urinary ACR |
| Fish | Park *et al.* 2019 | CARDIA study, USA | ●Women: 53%  ●Age: 25 ± 4y  ●Black: 50%  ●eGFR: 124 ± 16 ml/min/1.73m^2^  ●Fish intake  Non-fried fish: 0. ± 0.98 serving/day  Fried fish: 0.06 ± 0.27 serving/day | 489 / 4,133  **number of people with trace elements measured = 3,690** | Mean: 22.3y | Interview-based dietary history questionnaire | ●Incident CKD defined as eGFR <60 ml/min/1.73m^2^ or albuminuria >30 mg/g (urine ACR)  ●eGFR assessed with CKD-EPI_creatinine_ equation. ●Albuminuria determined from a single untimed urine sample | **For n=4,133**  Every serving per day increment  HR (95% CI) = 0.86 (0.73; 1.01)  **For n=3,690**  Every serving per day increment  HR_without adjustment_ (95% CI) =0.86 (0.73; 1.02)  HR_with adjustment_ (95% CI) =  0.86 (0.72; 1.02) | **For n=4,133**  ● age, sex, race study center  ● BMI  ● current smoker  ● alcohol use  ● PA  ● education  ● total energy  ● fried fish intake  ● personal kidney problems  **For n=3,690**  ● age, sex, race study center  ● BMI  ● current smoker  ● alcohol use  ● PA  ● education  ● total energy  ● fried fish intake  ● personal kidney problems  ● toenail measurements of mercury, cadmium, selenium |
| Fruit | Wen *et al.* 2018 | Village-based Handan Eye Study, China | ●Women: 55%  ●Age: 50 ± 11y  ●ACR: 7 [3;13] mg/g  ●Intake fresh fruit  Never or rarely: 35%  1-3 times/month: 34%  1-2 times/week: 22%  ≥3 times/week: 9% | 629 / 3,574 | Median: 5.6y | Self-reported questionnaire | ●Albuminuria defined as urinary ACR ≥30 mg/g  ●albuminuria assessed with spot urine sample | ≥3 times/week vs never or rarely  OR (95% CI)= 0.56 (0.38; 0.83)* | ● age, sex  ● BMI, waist circumference  ● smoking  ● alcohol use  ● PA  ● education  ● regular consumption of fresh vegetables  ● diabetes  ● CVD  ● total cholesterol, HDL-c, TGs  ● hypertension, SBP  ● anti-hypertensive drugs  ● eGFR, ACR ratio |
| Sugar-sweetened soda | Bomback *et al.* 2010 | Community-based ARIC Study, USA | ●Women: 55%  Age: 54 ± 6y  ●Race  White: 73%  Black: 27%  Other: 0.3%  ●eGFR: 92 ± 21 ml/min/1.73m^2^  ●Soda drinking  <1 soda/day: 82%  1 soda/day: 12%  >1 soda/day: 6% | 3,288 / 9,451 | Mean: 3.0y | Validated 66-item semiquantitative FFQ | ●Incident hyperuricemia defined as serum uric acid >5.7 mg/dl in women and >7.0 mg/dl in men | >1 soda/d vs <1 soda/d  OR (95% CI) = 1.17 (0.95; 1.43) | ● age, sex, ARIC-field center, race  ● BMI  ● current tobacco use  ● alcohol use  ● caffeine intake, animal protein intake  ● hypertension  ● renal function |
| Diet quality | Chang *et al.* 2013 | Community-based CARDIA Study, USA | ●Men: 47%  ●Age: 35 ± 4y  ●African-American race: 41%  ●ACR women: 4 [3; 7]  ●ACR men: 5 [4; 8] | 77 / 2,354 | Mean: 15.0y | Validated CARDIA dietary history FFQ | ●Incident microalbuminuria, defined as presence of race and sex-adjusted ACR ≥25 mg/g at 2 or more follow-up examinations  ●ACRs were obtained from spot urine samples at baseline and follow-up | **Diet quality**  Good vs poor  OR (95% CI) =  0.5 (0.29; 0.91)* | ● age, sex, race  ● obesity  ● education  ● total energy intake  ● diabetes  ● family history of kidney disease  ● hypertension  ● baseline ACR |

*Indicates statistical significance. HR = hazard ratio; OR = odds ratio; CI = confidence interval; USA = United States of America; eGFR = estimated glomerular filtration rate; PA = physical activity; WHR = waist-hip ratio; ACR = albumin-creatinine; CARDIA = Coronary Artery Risk Development in Young Adults; BMI = body mass index; CVD = cardiovascular disease; SBP = systolic blood pressure; HDL-c = high-density lipoprotein-cholesterol; TG = triglycerides; ARIC = Atherosclerosis Risk in Communities.
